# Recent Advances on Sorting Methods of High-Throughput Droplet-Based Microfluidics in Enzyme Directed Evolution

**DOI:** 10.3389/fchem.2021.666867

**Published:** 2021-04-23

**Authors:** Xiaozhi Fu, Yueying Zhang, Qiang Xu, Xiaomeng Sun, Fanda Meng

**Affiliations:** ^1^Department of Biology and Biological Engineering, Chalmers University of Technology, Gothenburg, Sweden; ^2^Department of Clinical Laboratory Medicine, The First Affiliated Hospital of Shandong First Medical University & Shandong Provincial Qianfoshan Hospital, Shandong Medicine and Health Key Laboratory of Laboratory Medicine, Jinan, China; ^3^School of Basic Medicine, Shandong First Medical University & Shandong Academy of Medical Sciences, Jinan, China; ^4^Department of Chemistry and Chemical Engineering, Chalmers University of Technology, Gothenburg, Sweden

**Keywords:** sorting methods, enzyme directed evolution, microfluidics, droplet, high-throughput

## Abstract

Droplet-based microfluidics has been widely applied in enzyme directed evolution (DE), in either cell or cell-free system, due to its low cost and high throughput. As the isolation principles are based on the labeled or label-free characteristics in the droplets, sorting method contributes mostly to the efficiency of the whole system. Fluorescence-activated droplet sorting (FADS) is the mostly applied labeled method but faces challenges of target enzyme scope. Label-free sorting methods show potential to greatly broaden the microfluidic application range. Here, we review the developments of droplet sorting methods through a comprehensive literature survey, including labeled detections [FADS and absorbance-activated droplet sorting (AADS)] and label-free detections [electrochemical-based droplet sorting (ECDS), mass-activated droplet sorting (MADS), Raman-activated droplet sorting (RADS), and nuclear magnetic resonance-based droplet sorting (NMR-DS)]. We highlight recent cases in the last 5 years in which novel enzymes or highly efficient variants are generated by microfluidic DE. In addition, the advantages and challenges of different sorting methods are briefly discussed to provide an outlook for future applications in enzyme DE.

## Introduction

Nature itself is a great reservoir of various enzymes whose catalysis of substrates makes life and industry possible. Many ancestral enzymes have low catalytic efficiency and low specificity but might go through rounds of mutations and natural selection toward more specific and efficient variants. This process might take millions of years, which is part of natural evolution. Over the recent two decades, scientists are trying to mimic natural selection conditions in the laboratories and accelerate the selection toward desirable properties, which is called directed evolution (DE). The whole process starts from a mutant library of one existing enzyme, or *de novo* synthetic enzyme; and the mutant library could be generated by rational/semi-rational design or random mutagenesis. The variants are expressed *in vivo*/*in vitro* or in cell-free system and then selected for improved properties. An effective assay requires tight linkage of genotype and phenotype, so that promising variants could be subjected to further cycles of optimization (Zeymer and Hilvert, 2018).

High-throughput screening (HTS) is commonly defined as screening no <100,000 samples per day (Attene-Ramos et al., 2014), which equals to 1.16 test per second, i.e., 1.16 Hz. Traditional HTS is performed with microliter plates (MTPs) in 96-, 384-, or 1,536-well formats and agar plates whose throughput is ~10^4^ variants with manual operation or ~10^6^ variants with robots per day (Markel et al., 2020). While libraries of 10^10^ variants could be easily generated by error-prone PCR (You and Percival Zhang, 2012), traditional screening process is still time-consuming and labor-intensive. Automated fluorescence measurement and robotic colony picking lighten the tedious screening workload, but the physical and material constraints associated with spatial separation inherently limit throughput (Packer and Liu, 2015) and always come with significantly increased reagent consumption (Martis et al., 2011). The developments of microfluidics with integrated droplet generation, droplet manipulation, and screening modules make automation possible for the whole enzyme screening process. By inputting a library of enzyme variants, researchers could collect outputs of desired ones from up to 10^8^ candidates per day, while consuming 10^6^-fold less sample volume (Vallejo et al., 2019).

**Figure 1:**
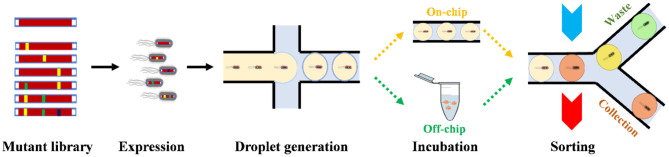
Typical microfluidic droplet workflow for enzyme directed evolution.

In a typical microfluidic droplet workflow (Figure 1) for enzyme DE, a single cell from the mutant enzyme-expression library is encapsulated in a water-in-oil (w/o) droplet with their substrates. The droplets could be incubated for a specific time for the enzyme to fully react with the substrate. Both on-chip and off-chip incubations are possible. If the enzymes are expressed *in vivo*, cell lysis buffer will also be encapsulated into the droplet in the droplet generation step. Then the droplets could be sorted based on specific detectable signals. According to the signals, dielectrophoresis (DEP) usually drags droplets with active enzymes inside toward a sorting channel by high-voltage electric pulses. The sorting methods determine the selection threshold for DEP at the junction. Many researches have been done in droplet generation step in the last decade, including w/o droplets (Bransky et al., 2009; Tarchichi et al., 2013), w/o-in-water (w/o/w) droplets (Nabavi et al., 2015), and hydrogel beads (Um et al., 2008; Marquis et al., 2015) from picoliter to nanoliter volumes; recently, there emerges rapid development of different sorting methods, along with practical applications in enzyme DE.

Since droplet generation frequency could reach 10~30 kHz, which means ~10,000–30,000 droplets per second, the efficiency of droplet sorting limits the efficiency of the whole microfluidics throughput. The sorting method constrains limits for droplet size and substrate concentration and affects the droplet recovery viability. Traditional microfluidic-based droplet sorting mainly relies on laser-induced fluorescence (LIF) detection. However, there have emerged other novel sorting methods for on-chip droplet HTS sorting recently and shows the trend of label-free sorting. In this work, both traditional labeled sorting methods and new label-free approaches in the past 5 years have been reviewed.

## Labeled Sorting

Labeled sorting is defined as sorting methods based on characteristics that do not result directly from the enzymatic reaction itself. Chemicals or tagged groups are added into the reaction so that labeled sorting methods could work on them or their derivatives. Fluorescence-activated droplet sorting (FADS) and absorbance-activated droplet sorting (AADS) are two commonly applied labeled sorting methods and also regarded as optical sorting. FADS relies on the fluorophore yield or fluorescent tagging in the droplet. AADS is based on changes in UV or visible light absorption. Its absorption changes are always caused by absorbing reagent yield in the enzymatic reaction or in its coupled assays.

### Fluorescence-Activated Droplet Sorting

FADS is based on a very similar principle to fluorescence-activated cell sorting (FACS). FACS has been considered as the gold standard for single cell sorting (Attene-Ramos et al., 2014) and could also be used for droplet sorting. Compared with FACS, FADS is performed on-chip and advantageous in the following aspects. Firstly, only hydrophilic w/o/w droplets could be sorted by FACS, while FADS could detect both w/o or oil in water (o/w) droplets and double emulsions. Secondly, high-speed camera makes it possible to visualize each droplet sorting event, which is not yet possible with FACS. Lastly, FADS devices are much cheaper than the dedicated FACS instrument.

FADS has been used for DE of aldolases (Obexer et al., 2017), DNA polymerases (Vallejo et al., 2019), NAD(P)-dependent oxidoreductases (Oyobiki et al., 2014), xylanase (Ma et al., 2019), lipases (Qiao et al., 2018), and oxidase (Debon et al., 2019) in bacteria (Qiao et al., 2018; Vallejo et al., 2019), yeast (Ma et al., 2019), and even filamentous fungi (Beneyton et al., 2016). It proves to be a powerful tool for enzyme DE.

**Figure 2:**
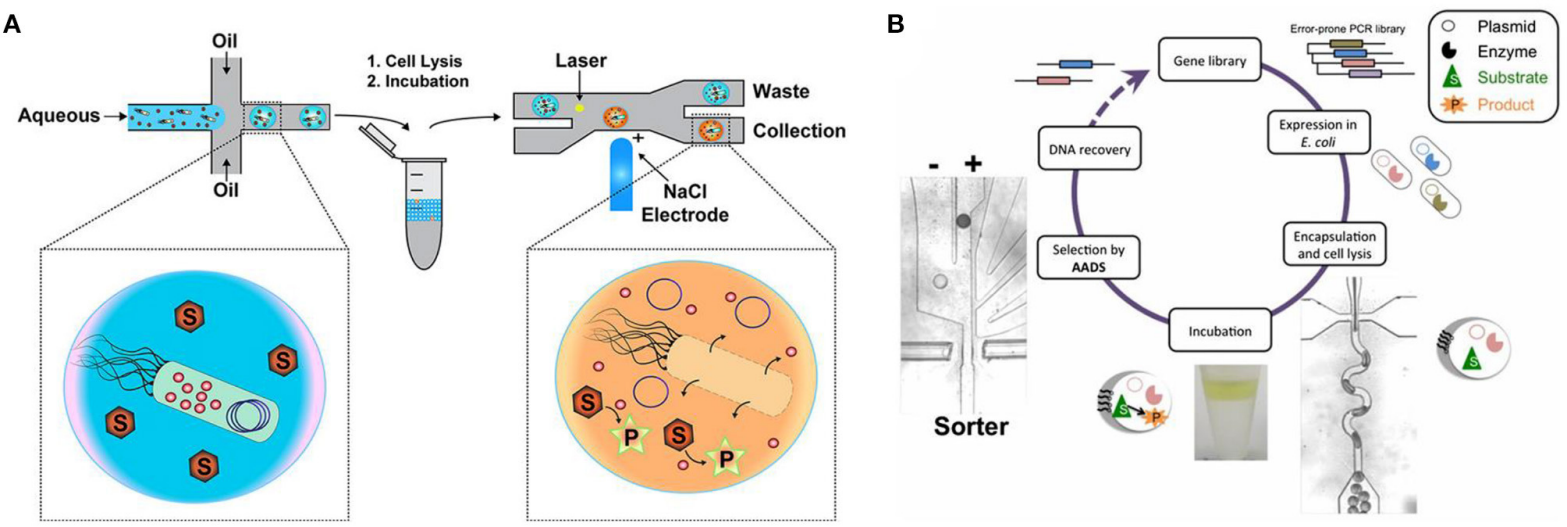
Labeled sorting methods based on fluorescence-activated droplet sorting (FADS) **(A)** [adapted with permission from Vallejo et al. (2019) Copyright © American Chemical Society] and absorbance-activated droplet sorting (AADS) **(B)** [adapted with permission from Gielen et al. (2016) Copyright © National Academy of Sciences].

In most cases, fluorogenic reporter substrate is needed for FADS. Ma et al. (2019) utilized the conversion of fluorogenic substrate 6,8-difluoro-4-methylumbelliferyl b-d-xylobiose (DiFMUX2) to fluorophore DiFMU by xylanase to evolve xylanase-producing *Pichia pastoris* and screened out a 1.3-fold mutant. Fenneteau et al. (2017) synthesized a new sulfonylated rhodamine for more sensitive peptidase activity detection with droplet-based microfluidics, so that absorption and emission ranges of yield product are separated. Larsen et al. (2016) found that Cy3-Iowa Black fluorophore–quencher pair for DNA labeling maintains a higher signal-to-noise ratio than commonly used fluorophore–quencher pairs. Vallejo et al. (2019) further applied 5′-Cy3 DNA labeling in the DE of nucleic acid enzymes, like polymerase, T4 ligase, and restriction enzyme with FADS. Vallejo et al. (2019, 2020) summarized this strategy as droplet-based optical polymerase sorting (DrOPS) (Figure 2A). Polymerase variant library is expressed in *Escherichia coli*, and single bacteria cells are encapsulated in microfluidic droplets. The polymerase is released into the droplet microcompartment upon cell lysis and will produce Cy3-based fluorescence signals by disrupting a donor–quencher pair. Fluorescent droplets with active polymerase variants could be sorted by FADS, and the plasmid encoding the variant could be recovered.

Instead of using fluorophore-labeling substrate directly, in other cases, researchers design secondary or tertiary reactions for the generation reporter chemicals of particular excitation/emission patterns. Debon et al. (2019) obtained a cyclohexylamine oxidase (CHAO) variant of 960-fold improvement compared with the wild type. In their case, amines are converted into imine by CHAO, meanwhile reducing 1 equiv. of flavin adenine dinucleotide (FAD). Oxygen-dependent cofactor recycling produces equimolar amounts of hydrogen peroxide (H_2_O_2_), which is detected by the downstream oxidation of a fluorogenic dye Amplex by horseradish peroxidase. This coupled-enzyme assay is applicable to other oxidases or relative enzymes, like l-asparaginase (Lim and Gruner, 2020) and glucose oxidase (Prodanović et al., 2020), which produced H_2_O_2_ and secondary reactions were added so that fluorophore was further produced.

Researchers have developed some upgraded versions of commonly used FADS system. Two fluorescence-activated droplet sorters were set in series along the chip and equipped with two sets of excitation/emission wavelengths (Ma et al., 2018). A double-gated control algorithm could process both fluorescence signals from the same droplets. Two chemical reactions happened simultaneously in one droplet so that esterase mutants from *Archaeoglobus fulgidus* with both high enzymatic activity and high enantioselectivity could be sorted.

Researchers also look into other characteristics of fluorescence and newly developed fluorescence lifetime-activated droplet sorting (FLADS) (Hasan et al., 2019) based on the lifetime of fluorophore. FLADS has been successfully applied to distinguish droplets containing either pyronine or fluorescein, or both, since both chemicals are excited at the wavelength of 470 nm (Hasan et al., 2019) but have different fluorescence lifetime. This might help broaden the scope of fluorescence sorting but presently is limited by the throughput, whose frequency of only up to 50 Hz was achieved till now (Haidas et al., 2019).

### Absorbance-Activated Droplet Sorting

AADS helps extend the application of microfluidics, by breaking the exclusive boundary of fluorescent readouts. Gielen et al. (2016) firstly set up an AADS coupled droplet microfluidic for enzyme DE (Figure 2B), by embedding two optical fibers aligned face-to-face across the droplet channel. With this device, the activity of phenylalanine dehydrogenase *Rhodococcus* sp. M4 was improved >4.5-fold in lysate and k_cat_ increased >2.7-fold after two rounds of DE. The reduction of the cofactor NAD^+^ to NADH in the deamination direction is detected by a coupled assay involving the electron coupling reagent 1-methoxy-5-methylphenazinium methyl sulfate (mPMS) and the reduction of the water-soluble tetrazolium salt 2-(4-iodophenyl)-3-(4-nitrophenyl)-5-(2,4-disulfophenyl)-2H-tetrazolium (WST-1) to give the absorbing dye WST-1 formazan. The sorting frequency could achieve ~1 million droplets per hour. Theoretically, AADS could have much more applications since most small molecules exhibit absorption in the UV and visible regions of electromagnetic spectrum.

AADS has the inert disadvantage of reduced optical pathlength together; its sensitivity is three to four magnitude lower than that of FADS (Table 1). To address the sensitivity problem, Maceiczyk et al. (2017) combined differential detection photothermal interferometry (DDPI) with absorbance detection in droplet-based microfluidics. DDPI allows for quantitative, single-point absorbance detection in femtoliter–picoliter volume droplets and is weakly dependent on the optical pathlength. They applied this method to detect 100 pl with as low as 1.4 μM of erythrosine B at 1-kHz frequency and femtoliter droplets at 10-kHz frequency. This method was proved workable for colorimetric assay of HL-60 cells growth and β-galactosidase activity, but it has not been applied in enzyme DE yet.

**Table 1:** Optimal specification of different sorting methods.

**Sorting methods**	**Sensitivity**	**Highest frequency**	**Minimal droplet size**
FADS	2.5 nM (Colin et al., 2015)	5 kHz (Neun et al., 2019)	2 pl (Colin et al., 2015)
AADS	10 μM (Gielen et al., 2016)	100 Hz (Gielen et al., 2016)	100 pl (Maceiczyk et al., 2017)
ECDS	1 μM (Goto et al., 2020)	10 Hz (Goto et al., 2020)	30 nl (Goto et al., 2020)
MADS	5 μM (Kempa et al., 2020)	35 Hz (Kempa et al., 2020)	0.8 nl (Kempa et al., 2020)
RADS	200 μM (Sobota et al., 2019)	4.3 Hz (Wang et al., 2017)	65 pl (Wang et al., 2017)
NMR-DS	1 mM (Davoodi et al., 2020)	—	130 pl (Hale et al., 2018)

—*means not reported*.

Instead of improving the sensitivity of AADS itself, Zurek et al. (2021) recently developed a strategy by increasing enzyme molecules in the droplets. They set up a workflow for clonal amplification in droplets and demonstrated that around 400 *E. coli* cells will be in one 100-pl droplet after single-cell cultivation overnight. Through increasing enzyme molecules, the reaction rate of phenylalanine dehydrogenases (PheDH) improved 12-fold as detected in absorbance assay of droplets. The same strategy might also be applied for other less sensitive sorting methods.

## Label-Free Sorting

Apart from the two common optical (labeled) sorting approaches, a trend of developing label-free detections emerged, which uses intrinsic physical or chemical biomarkers to separate and sort cells. Several major label-free sorting methods are like electrochemical detection, mass spectrometry (MS), and Raman and nuclear magnetic resonance (NMR) coupling with a microfluidic chip. Different from easy coupling of optical sorting set up with microfluidic devices, the following label-free sorting methods often need a specific design or coupling techniques.

### Electrochemical-Based Droplet Sorting

Electrochemical detection is label free and can be applied to complex samples without interference from suspension, autofluorescence, or staining. Due to the limitation of detection electrode surface size, which should not be larger than the droplet, the size of droplets applied is normally limited to nanoliter. The detection device on a microfluidic chip could also be coupled with a DEP sorting device, by converting electrochemical signal into digital signal and then into alternating current (AC) signal to turn on DEP sorter.

**Figure 3:**
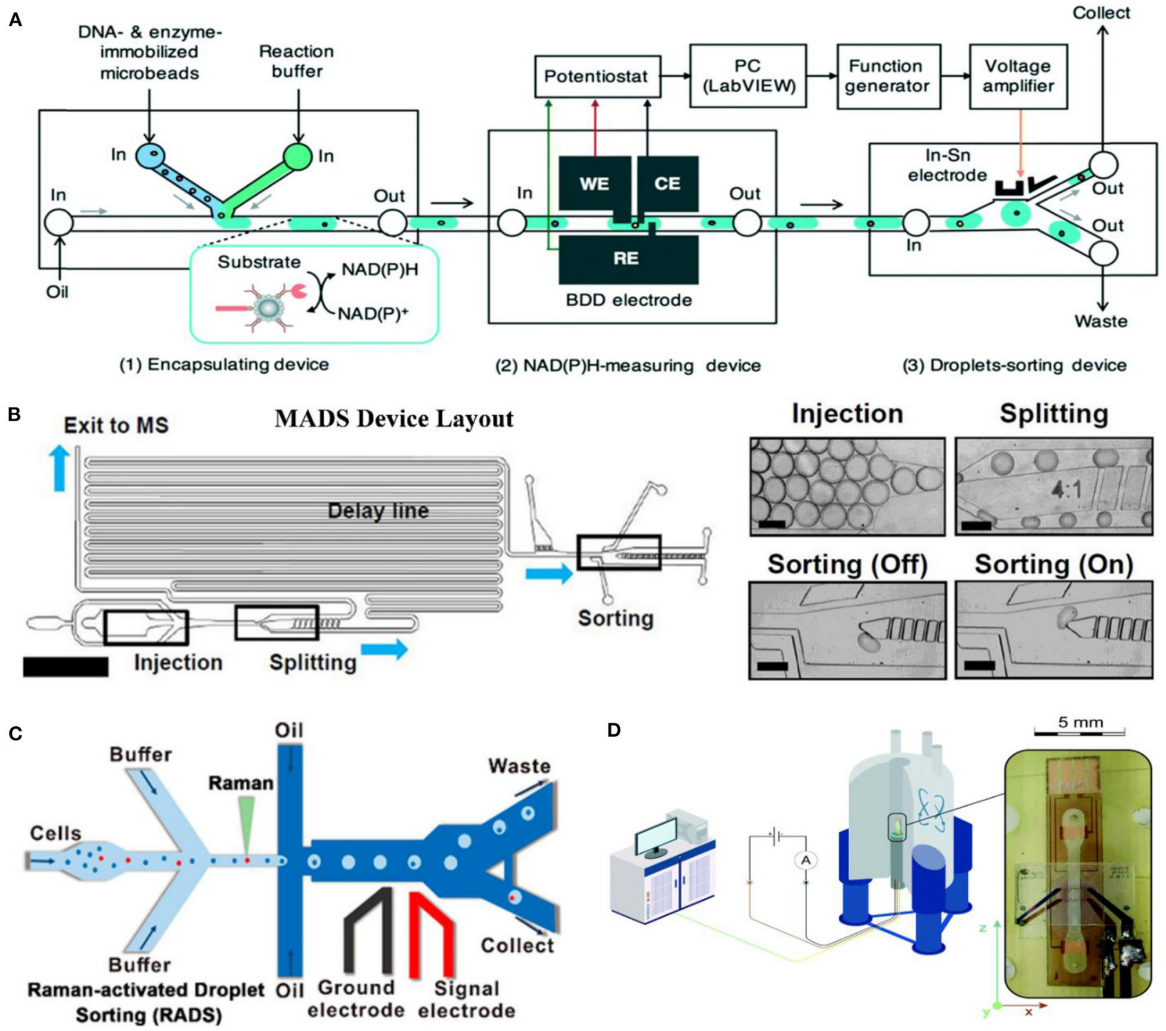
Label-free sorting methods based on electrochemical detection **(A)** [adapted with permission from Goto et al. (2020) Copyright © Royal Society of Chemistry], mass spectrometry **(B)** [adapted with permission from Holland-Moritz et al. (2020) Copyright © John Wiley and Sons, Inc.], Raman **(C)** [adapted with permission from Wang et al. (2017) Copyright © American Chemical Society], and NMR **(D)** [adapted with permission from Davoodi et al. (2020) Copyright © Royal Society of Chemistry].

Goto et al. (2020) applied a boron-doped diamond (BDD) electrode to measure the current change in 30-nl droplets for the DE of a NAD(P)-dependent oxidoreductase. As low as 1 μM of NADH could be detected, and a 3-fold higher activity of isocitrate dehydrogenase (IDH) mutant from *Streptococcus* mutants was screened (Figure 3A). Oyobiki et al. (2014) proved previously that the characteristics of BDD, such as wide potential window, a low background current, and the higher stability against deactivation, are suitable for measuring NADH oxidation.

A Sorting based on Interfacial Tension (SIFT) method was developed even without extra electrodes to differentiate droplets of various interfacial tension due to different pH values in droplets (Abbyad et al., 2019). Low pH leads to high interfacial tension of droplets, and droplets of lower pH tend to flow along a trail on a chip. The method has been developed to distinguish between pH 0.2, and maximum sorting rate is around 30 Hz (Horvath et al., 2019). However, this method has not been applied to any enzyme high-throughput sorting yet.

### Mass-Activated Droplet Sorting

As the most widespread and versatile analytical technique to analyze chemical mixtures, MS has been combined with microfluidics in recent 5 years. Both electrospray ionization (ESI) (Mahler et al., 2018; Qiao et al., 2018; Kempa et al., 2020) and matrix-assisted laser desorption ionization (MALDI) (Haidas et al., 2019) have been applied in droplet HTS. ESI could produce charged ions directly from a liquid, which facilitates it to be coupled with microfluidics. However, the online coupling of MALDI with microfluidics is challenging, because MALDI target is under vacuum while the microfluidic droplets are generated under atmospheric pressure. Meanwhile, MALDI cannot be performed as an online technique for analysis, which also restricts its throughput (Payne et al., 2020).

Oil carrier phase usually needs to be removed before directing the aqueous flow into an electrospray emitter, since oil phase interferes with the ESI process by both sequestering charge carriers and preventing stable Taylor cone formation. An orthogonal chip-MS set up (Beulig et al., 2017) was developed by placing a grounded metal plate as a counter-electrode with a specific distance to the spray on the chip, while orthogonal to the MS orifice. The inlet flow to MS orifice could be adjusted by changing the distance from MS orifice to the spray. This setup could avoid sample overloading while maintaining a stable electrospray. Sample overloading might cause transfer line contamination and signal saturation.

Researchers also developed another strategy of applying a specific rate of sheath flow so that the whole w/o droplets could be directly infused into ESI (Diefenbach et al., 2018; Holland-Moritz et al., 2020). Carryover could be eliminated by replacing hydrophilic stainless (SS) needle with Teflon ESI needle to avoid cross contamination between droplets (Diefenbach et al., 2018).

Since MS detection is sample disruptive (Haidas et al., 2019), MS is usually coupled with microfluidic for enzymatic catalysis analysis. Holland-Moritz et al. (2020) developed a mass-activated droplet sorting (MADS) system based on ESI (Figure 3B). In their system, 25-nl droplets are split into two portions by a T-split line on a chip almost asymmetrically. A 15-nl daughter droplet flows directly into perfluoroalkoxy alkane (PFA) capillary for quadrupole mass spectrometer via a sheath-flow ESI source. The other daughter droplet is sorted by DEP. Sorting decisions are made based on the MS signals and time delay between these two daughter droplets. This system achieved 0.7 samples per second with 98% accuracy in transaminase ATA-117 cell-free system.

The MS detection rate is normally around 0.5–1.0 s per droplet (Oyobiki et al., 2014; Mahler et al., 2018) and requires nanoliter-sized droplets (Wink et al., 2018; Holland-Moritz et al., 2020). Up to 33-Hz nanosized droplet sorting rate was achieved by Kempa et al. (2020), by coupling traveling-wave ion mobility quadrupole time of flight (TWIMS Q-TOF) with microfluidics. Such on-chip MS detection has been applied in chemical reaction analysis, either with cells (Diefenbach et al., 2018; Mahler et al., 2018) or not (Beulig et al., 2017).

### Raman-Activated Droplet Sorting

Unlike MS, RADS is non-invasive but also label-free. RADS could sort cells at a rate of hundreds of cells per minute [up to 500 cells/h achieved (Lee et al., 2020a)]. Wang et al. (2017) (Figure 3C) developed RADS platform by placing single-cell Raman spectrum (SCRS) prior to droplet generation, since Raman background of oil medium might adversely affect the system's accuracy. Single cells are detected by SCRS, encapsulated into w/o droplet and then directly flow to DEP sorting. This system was successfully applied to screen out *Haematococcus pluvialis* with high astaxanthin yield and a relatively high throughput (~260 cells/min) and high accuracy (~98%) were achieved.

Most Raman-based sorting assays are based on resonance signals. Mcilvenna et al. (2016) reported continuous sorting of cyanobacteria based on carotenoids with Raman-microfluidic system. By adding ^13^C-bicarbonate into the culturing medium, shifts in carotenoid bands could be measured, indicating active dissolved-CO_2_-fixing cells. Lee et al. (2020b) prepared a D_2_O-containing minimal medium supplemented with unlabeled interested compound mucin and detected the changes of cytochrome signal with Raman-based microfluidics. Mucin-utilizing Muribaculaceae strains were successfully screened out from a mouse with this device.

Resonance signals however associate with only a few classes of cellular compounds like pigments and therefore limit the application genotype scope. Actually, Raman spectrum is informative in not only resonance signals but also non-resonance ones. Non-resonance signals associate with more chemicals *in vivo* (e.g., starch, protein, and nucleic acid); nevertheless, they need longer acquisition time, which usually conflicts with throughput.

Recent breakthroughs have just been made in developing non-resonance-based RADS. A significantly improved rate of 120 cells/min and two variants of an unknown enzyme [algal diacylglycerol acyltransferases (DGATs)] were discovered by applying this RADS setup (Wang et al., 2020). A quartz-made chip, with low background signals, could be used for non-resonance signal RADS rather than PDMS.

The major disadvantage of Raman spectrometry is its relative low sensitivity. Researchers make attempts by fabricating surface-enhanced Raman spectrometry (SERS) substrates to improve the sensitivity. Sobota et al. (2019) applied a SERS substrate SK307 and detected as low as 200 μM of 1,2,3-trichloropropane on SERS-coupled microfluidics, which might inspire the screening of haloalkane dehalogenase enzymes.

### NMR-Based Droplet Sorting

NMR could give information from all states of matter in a wide range of temperatures. Unlike MS, NMR offers an option of a completely non-invasive metabolomic readout. Theoretically, NMR is obviously disadvantageous in its low intrinsic mass sensitivity, which means normal concentrations below 1 mM are hard to observe (Davoodi et al., 2020). Davoodi et al. (2020) developed an NMR-compatible microfluidic platform by placing the metal tracks in the side walls of a microfluidic channel (Figure 3D). NMR radio-frequency excitation performance was found to be actually enhanced without compromising B_0_ homogeneity.

For NMR, pre-shimming of samples is significant, so that homogeneity and stability of the magnetic field could be obtained. Shimming is usually performed by applying currents to various shim coils. With the design of highly efficient planar NMR Helmholtz microcoil and transmission line resonators, the problem of the NMR sensitivity on small volume samples could be solved. Van Meerten et al. (2018) simplified the regular shim coils with a series of parallel wires placed perpendicular to B_0_ as a Shim-on-Chip shim system, which is particularly suited for microliter samples in capillaries. To further address the challenge of interfacing microcoils with droplets, Lei et al. (2015) firstly reported interface between digital microfluidics and low-field NMR. Later, Swyer et al. (2016) developed the first digital microfluidic system capable of interfacing droplets of analyte with microcoils in high-field NMR, which is appropriate for chemical characterization. The system was successfully applied to monitor a glucose oxidase reaction in 4-μl droplets, but not for sorting yet.

Another challenge that hinders NMR application in microfluidics is the preservation of high spectral resolution, which requires a highly homogenous magnetic field over the sample volume. However, differences in magnetic susceptibility between the chip, the continuous phase, and the droplet phase will lead to a demagnetizing field that varies continuously over the sample volume (Hale et al., 2018). Researchers show that susceptibility difference between the chip and the continuous phase could be mitigated by a combination of structural shimming and doping of the less diamagnetic of the liquid phases with a europium compound, such as Eu^3+^ compound (Hale et al., 2018).

## Discussion and Conclusions

Droplet-based microfluidics is becoming a powerful toolbox for enzyme DE, especially for a randomly generated mutant library. There are some breakthroughs of highly efficient enzymes screened out by microfluidics. Droplets usually go through “generation,” “incubation,” “manipulation (optional),” and “sorting” steps on a chip. For the first three steps, there have been many technological advances in the last decade, and there have already been commercial droplet generation toolkits and droplet manipulation devices like a picoinjector (Abate et al., 2010). More generally, droplets could be generated at several to tens of kHz frequency (Zhu and Wang, 2017). The major limitation lies in the sorting step, whose diversity and frequency restrict the sorting efficiency and target enzyme scope.

FADS is the most mature sorting method and reaches the highest sorting rate in all sorting methods. FADS is also of highest sensitivity and could be as low as 2.5 nM of fluorescein at a 2-pl droplet (Hasan et al., 2019), which means less than a single turnover for all enzyme molecules from a single cell (Table 1). Genotype is usually linked with fluorescent phenotype by fluorophore activation (Qiao et al., 2018), quencher release (Nikoomanzar et al., 2019), or coupled assay. In general, FADS is the primary choice if fluorescence could be achieved in the enzymatic reaction. AADS is similar with FADS, even if of lower efficiency and sensitivity, which could offer another option for broader enzyme applications. AADS and FADS could even be combined into one chip to be more informative. Siltanen et al. (2018) designed a FAADS (fluorescence and AADS) device in which a source-coupled fiber for excitation and two fibers for transmittance and emission were embedded on the chip. The excitation fiber is connected with continuous-wave lasers with 405, 473, 532, and 640 nm. Emitted light and transmitted light are collected by the other fibers, so that optical density and fluorescence values are obtained almost simultaneously.

Label-free sorting methods are drawing more and more attention, since they are advantageous in maintaining the integrity and independence of the whole enzymatic reaction system, away from any extra disruptions. What is more, they offer various options for researchers and save the trouble of linking the genotype with fluorescent phenotype. In principle, they all could be used for droplet screening and recycling for further gene recovery and sequencing. Even for MADS, splitting chip made it possible to detect and recycle droplets (Holland-Moritz et al., 2020). Challenges for broad applications of label-free detection lie in low sorting frequency and complex sorting device design. For electrochemical sorting, there has not been a standardized way for the sorting device and might even need to be customized according to various electrochemical characteristics. RADS and NMR-based sorting requires special techniques to set up the sorting system. Standardization in design and convenience for microfluidic coupling will be the future direction for label-free detection setup. Presently, there are just few application cases of label-free detection in enzyme DE.

When choosing among all sophisticated droplet-based sorting methods, we need to consider cell species, enzyme type, enzyme yield, enzymatic efficiency, etc. FADS and AADS would be the primary two choices if optical change could be linked in the enzymatic reaction. The droplet size for AADS needs to be carefully considered, since sufficient enzyme molecules need to be accumulated to stimulate the optical detection due to AADS's relative low sensitivity. Even though there are rare application cases, electrochemical-based sorting is promising in a wider application if the physical electrodes and electrochemical sensors are more versatile. MADS is efficient for low-concentration substrate and could be an option especially for isomers. Raman-activated cell sorting (RACS) would be a good choice if richer information is required apart from the enzymatic reaction itself (Wang et al., 2020); meanwhile, the relative low throughput can be compromised. Moreover, another informative sorting method-NMR-based sorting is molecule-informative while of low throughput (Hale et al., 2018). Table 1 shows the optimal specification in which different sorting methods have been achieved in recent publications.

Technically speaking, a sorting device includes a detector and a sorter. There are various sorters like acoustic, magnetic, pneumatic, thermal, and electric actuation sorters. Among them, DEP is the most widely used one. The typical DEP sorting setup consists of a sorting junction linked by two-way channels. Without an electric field, droplets would be sent to a waste output channel. Otherwise, an electric field is triggered by the detection part, and positive droplets could be sent to the collection channel by adjusting the hydrodynamic resistance (higher than that of the waste channel) (Frenzel and Merten, 2017).

There are also some attempts to extend traditional DEP sorter to multiple channels, so that the whole system could reach a higher efficiency. Frenzel and Merten (2017) designed a sorting module, in which four channels sequentially branch off from the waste channel and each collection channel has its own electrode pair running parallel to the channel wall. They achieved an ~100% reliable two-way sorting, largely independent of the relative flow rates in the channels downstream of the sorting junction. In another design (Caen et al., 2018), five channels were designed with the sorter, and droplets could be sorted by different voltages applied by two symmetrical live and ground electrodes. The device was successfully applied to screen droplets of different concentrations of a fluorescent dye sulforhodamine B and reached a sorting rate of up to 200 droplets per second (Caen et al., 2018). Combined with downstream next-generation sequencing, a multichannel could offer more information about variants and help accelerate the DE rate by deep learning. Those developments will help make droplet sorting for enzyme DE more versatile in the future.

## Outlook

DE of enzymes toward high specificity and efficiency is significant to both scientific researches and industrial applications. Droplet-based microfluidics paves a cheap and convenient way for enzyme DE with ultra-high throughput, and meanwhile, it is becoming a useful tool for *de novo* synthetic enzyme screening. Sorting methods, as the main step in DE, determine the efficiency of the whole system. Sorting devices are developing toward standardized modules compatible with different instruments and microfluidic chips. Combinations of different sorting methods could help gain multiplex perspectives into enzyme and boost a wide range of application.

## Author Contributions

XF and FM conceived the concept, conducted literature survey, and drafted and revised the manuscript. All the authors organized the figures and approved them for publication.

## Conflict of Interest

The authors declare that the research was conducted in the absence of any commercial or financial relationships that could be construed as a potential conflict of interest.
